# Insights into Theranostic Properties of Titanium Dioxide for Nanomedicine

**DOI:** 10.1007/s40820-019-0362-1

**Published:** 2020-01-14

**Authors:** Morteza Hasanzadeh Kafshgari, Wolfgang H. Goldmann

**Affiliations:** 1 Department of Engineering Physics, Polytechnique Montreál, Montreal, QC H3C3A7 Canada; 2 Department of Physics, Biophysics Group, University of Erlangen-Nuremberg, 91052 Erlangen, Germany

**Keywords:** TiO_2_ nanostructures, Drug delivery systems, Bioimaging, Biosensing, Tissue regeneration

## Abstract

Titanium dioxide (TiO_2_) nanostructures exhibit a broad range of theranostic properties that make them attractive for biomedical applications. TiO_2_ nanostructures promise to improve current theranostic strategies by leveraging the enhanced quantum confinement, thermal conversion, specific surface area, and surface activity. This review highlights certain important aspects of fabrication strategies, which are employed to generate multifunctional TiO_2_ nanostructures, while outlining post-fabrication techniques with an emphasis on their suitability for nanomedicine. The biodistribution, toxicity, biocompatibility, cellular adhesion, and endocytosis of these nanostructures, when exposed to biological microenvironments, are examined in regard to their geometry, size, and surface chemistry. The final section focuses on recent biomedical applications of TiO_2_ nanostructures, specifically evaluating therapeutic delivery, photodynamic and sonodynamic therapy, bioimaging, biosensing, tissue regeneration, as well as chronic wound healing.

## Highlights

- Multifunctional TiO_2_ nanostructures hold promise for advancing a wide range of biomedical applications due to a feasible integration of distinct theranostic features.
- Fabrication and post-fabrication strategies implemented to generate multifunctional TiO_2_ nanostructures for a broad range of biomedical applications are briefly outlined. The opportunities and challenges of TiO_2_ nanomaterials are highlighted in order to open the possibility of clinical translation.

## Graphical abstract

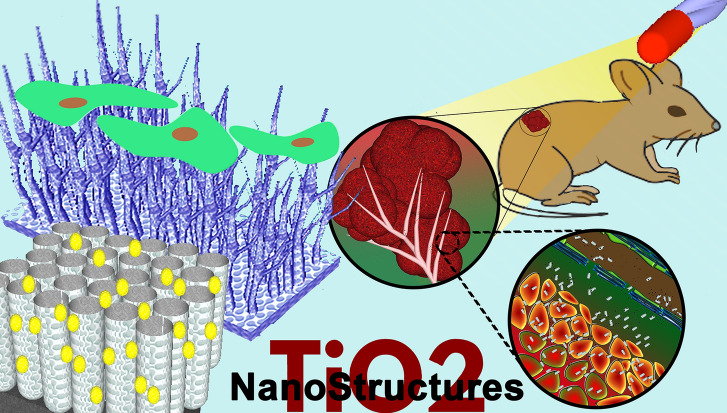

## Introduction

Titanium dioxide (TiO_2_) bulk materials are often employed in medical applications and devices, including implants, facial cosmetic surgeries, cardiovascular devices, external prostheses, as well as surgical instruments. When approaching nanoscale dimensions of bulk TiO_2_, quantum confinement occurs over superfine pieces and introduces new physical, mechanical, optical, and electronic properties [1, 2]. Compared to conventional bulk materials, TiO_2_ nanostructures (NSs), developed in different morphologies (i.e., sphere, tube, cylinder, fiber, sheet, whisker, wire, and rod) through feasible and reproducible fabrication strategies, have been employed in a wide range of leading-edge biomedical applications [2–6]. These efforts, for example, have resulted in enhancing drug delivery systems through the fabrication of porous TiO_2_ nanocarriers due to a huge surface-to-volume ratio, which can enlarge the therapeutic loading capacity [7–9]. The performance of TiO_2_ nanomaterials in biomedical applications can also depend on nanoscale morphologies and their specific properties. Besides their improved surface area, one-dimensional TiO_2_ nanocarriers designed to adhere more on the vascular endothelium compared to spherical nanoparticles at the tumor microenvironment, have ameliorated the cellular uptake and intracellular therapeutic delivery [10, 11]. To achieve the best performance, the fabrication of TiO_2_ nanomaterials with a well-designed composition, morphology, crystalline structure, and integration is an advantage.

Besides their intrinsic properties, an elaborated surface modification, such as a harmless doping, deposition, and biomolecule conjugation, can completely generate unique TiO_2_ nanomaterials with a specific biomedical application-oriented feature. The possibility of a thermal hydrogenation generating black TiO_2_ nanomaterials, a thermal oxidization altering crystalline structure or even a solvothermal method fabricating mesoporous TiO_2_ popcorn particles, can boost the photocatalyst activity compared to other nanomaterials (i.e., based on carbon or silicon) for photodynamic therapy [12–16]. Compared to other nanomaterials, TiO_2_ NSs can also be easily modified to become thermo-, pH-, X-ray-, and ultraviolet (UV)-responsive nanocarriers to advance drug delivery systems and eliminate such side effects of conventional chemotherapy [8, 10, 17–20]. In addition, accumulation of TiO_2_ nanomaterials at target tissues in the body can be become improved through a conjugation of biomolecules (i.e., folic acid and antibody) and deposition of iron oxide nanomaterials (i.e., magnetically guided therapeutic delivery) [8, 12, 21]. Biodistribution and accumulation of bare and surface-modified TiO_2_ nanomaterials in the body can also be visualized and verified using a magnetic resonance imaging (MRI) and fluorescence-based microscopy to accurately detect the target tissue prior to therapy in order to minimize side effects [22–25].

The detection (e.g., of circulating cancer cells and pathogens), as well as small biomolecules in clinical blood samples, has been advanced through the use of TiO_2_ platforms (i.e., label-free microfluidic immunosensors, photoelectrochemical biosensors, field-effect transistors, and amperometrics) [26–30]. To improve the detection performance, the band gap engineering of TiO_2_ NSs using a simple doping or deposition process is highly feasible and efficient compared to other nanoplatforms [31, 32]. Therefore, reusable and high-precision biosensors are highly likely to enter the market with the aid of enhanced cost-effective TiO_2_ nanomaterials, which possess a wide band gap and high surface activity [2, 29].

TiO_2_ nanomaterials are biocompatible and less toxic than other nanomaterials (i.e., copper oxide, zinc oxide, and manganese oxide) [33, 34]. The long-term stability of TiO_2_ nanomaterials in biological conditions is another advantage that can protect the loaded biomolecules from denaturation in comparison to other unstable (degradable) nanomaterials in an aqueous solution (i.e., a fast dissolution of silicon-based nanomaterials can quickly disassociate and release the loaded therapeutics) [19]. Besides their biocompatibility and stability, TiO_2_ NSs are also well known for tissue regeneration owing to high tensile strength, flexibility, corrosion resistance, as well as cellular adherence and proliferation [35–38]. Moreover, the photocatalytic activity of TiO_2_ nanomaterials is another advantage used to fight against antibiotic resistant bacteria in order to accelerate chronic wound healing by enhancing cellular adhesion and proliferation [39, 40].

In this review, we re-examine advanced strategies for the formation of TiO_2_ nanomaterials and present a summary of post-fabrication and surface chemistry approaches developed to generate elaborated TiO_2_ nanoplatforms for a broad range of biomedical applications. We briefly discuss biological responses following the administration of bare and surface-modified TiO_2_ nanomaterials in vitro and in vivo to highlight possible induced cytotoxicities and inflammations. We further delineate recent research achievements in therapy, diagnostic biosensing, tissue regeneration, and wound healing in vitro and in vivo, and pay attention to the developed TiO_2_ nanoplatforms for biomedical applications and address opportunities to initiate next-generation technologies and cutting-edge nanoscale devices.

## Fabrication of TiO_2_ NSs

The fabrication of TiO_2_ NSs can be broadly classified as bottom-up (an individual element progresses through homogeneous nucleation and growth) and top-down processes (the successive fragmentation of a bulk material into a series of nanoscale structures) [1–3]. The most common TiO_2_NSs (listed below) can be fabricated by both bottom-up and top-down strategies to introduce a specific theranostic feature for biomedical applications. Strategies are summarized in this section that overviews the development of mono- and multifunctional TiO_2_ NSs for nanomedicine.

### Nanoparticles

TiO_2_ nanoparticles are the most common NSs widely employed for nanomedicine. An inexpensive mass production of pharmaceutical TiO_2_ nanoparticles with a narrow size distribution, adjustable polymorphism, and surface property can feasibly accelerate their use for biomedical applications such as therapeutic delivery and diagnosis [41]. Bottom-up techniques, including sono-chemical strategies, hydrothermal approaches, microwave processes, chemical/physical vapor deposition, microemulsion, and sol–gel techniques, have been mostly applied to generate narrow-sized TiO_2_ nanoparticles with a flexible surface chemistry in comparison to the top-down processes [1, 41, 42].

For therapeutic delivery, the formation of nanoscale TiO_2_ with a high surface area (i.e., 587.7 m^2^ g^−1^ for particles 9 nm in diameter [43]) and porous structure are the compulsory properties [44]. Inducing porosity within the structure of TiO_2_ nanoparticles can increase the specific surface area. An adjustable pore size from a couple of nanometers to a few nanometers is beneficial for packing a wide range of therapeutic agents [45]. For example, mesoporous TiO_2_ nanoparticles were prepared through a surfactant-assisted hydrometallurgical procedure of ilmenite concentrate, and the pore size of the porous particles (around 30–60 nm in diameter) could be varied from 2 to 12 nm [46].

**Fig. 1:**
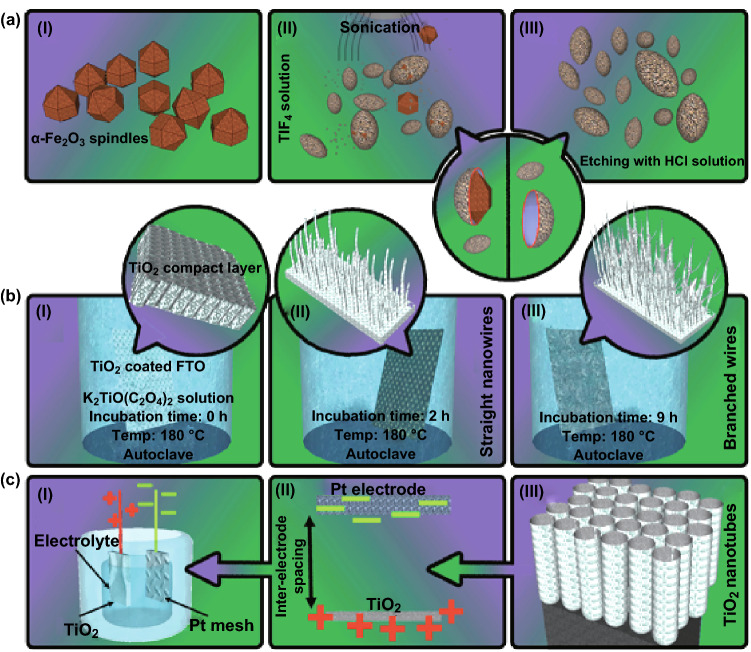
Simplified schematic representation of fabrication techniques for TiO_2_ nanomaterials. **a** Non-spherical, hollow, and magnetically loaded particles fabricated through a template-assisted, bottom-up strategy. Adapted from Ref. [49] with permission from the John Wiley & Sons. **b** The hydrothermal approach is one of the bottom-up strategies for the fabrication of a wide range of one-dimensional TiO_2_ NSs. The TiO_2_ precursor, temperature, and incubation time determine the final nanostructure. Adapted from Ref. [55] with permission from the Springer Nature. **c** Well-aligned and ordered TiO_2_ nanotubes can be fabricated through a top-down strategy based on electrochemical anodization. Adapted from Ref. [74] with permission from the American Chemical Society

Multifunctional nanoparticles, which are employed in targeted drug delivery systems and photodynamic therapy, are the most common structure developed in this category. Multifunctional TiO_2_ nanoparticles with a magnetic core are a favorite nanohybrid for implanting a wide range of theranostic features, which include magnetic-guided and triggered therapeutic delivery systems [47, 48]. For example, mesoporous TiO_2_-coated Fe_3_O_4_ nanoparticles have recently been developed through a combined fabrication strategy, i.e., the solvent thermal method to generate an amino-functional magnetic core and homogeneous precipitation of TiOSO_4_ to form a porous shell [48]. To generate hollow TiO_2_ nanoparticles, fabrication of iron oxide TiO_2_ core–shell nanocomposites is an advantage due to the easy removal of the magnetic core within the process. As shown in Fig. 1a, a homogeneous deposition of anatase TiO_2_ onto α-Fe_2_O_3_ nanotemplates forms core–shell nanoparticles, and a subsequent etching procedure (HCl 0.2 M at 100 °C for 6–24 h) removes the core template, resulting in a hollow structure [49]. A wide range of multifunctional nanoparticles can also be produced through the combination of a couple of approaches, including the template-assisted technique and hydrothermal strategy for providing an individual crystalline phase, polymorphism, size distribution, and porosity in situ [50, 51]. Multifunctional polypyrrole-coated mesoporous TiO_2_ nanocomposites, for example, can be fabricated through pre-hydrolysis of titanium precursors combined with the solvothermal treatment strategy for photothermal, sonodynamic, and chemotherapeutic treatments and dual-modal ultrasound/photoacoustic imaging of tumors [7, 52].

### Nanowires and Nanorods

One-dimensional (1D) TiO_2_ nanowires and nanorods are one of the powerful platforms that play a critical role in capturing and transmitting the biological responses at the interface required for the development (e.g., of ultrasensitive detection devices) [53, 54]. 1D TiO_2_ nanowires and nanorods have been synthesized by means of template-assisted methods and solution- or vapor-based approaches. Among these approaches, solution-based fabrication of TiO_2_ nanowires and nanorods is well known due to an easy mass production and desirable growth length and properties. Generation of a supercritical fluid at a specific temperature and pressure dissolves almost all solid TiO_2_ precursors, followed by a precipitation process to form nanowires or nanorods [2]. TiO_2_ nanowire arrays can be generated using a substrate precoated with TiO_2_ nanoparticles via the hydrothermal method [55]. To grow long TiO_2_ nanowire trunks with numerous short nanorod branches by a surfactant-free procedure, a mixed homogeneous solution (K_2_TiO(C_2_O_4_)_2_, diethylene glycol and water) can be initially poured into a Teflon-lined stainless steel autoclave with FTO glass and then heated up to 180 °C using a hydrothermal method for 1–12 h (Fig. 1b). The fabrication of branch-type TiO_2_ nanowires, whether anatase or rutile, can also be created by hydrothermal processes [2]. The mechanisms involved in the fast growth of one-dimensional TiO_2_NSs through the self-assembly, require the crystal structure with superior anisotropic properties [56]. In the case of poor anisotropic TiO_2_, the self-assembly rate should be accelerated by introducing a precipitation interface, dislocation propagation direction, and higher constructive block concentrations [2]. The strategies and mechanisms involved in the fabrication of nanorods are largely similar to that of nanowires; however, nanorods are shorter while reflecting a smaller aspect ratio and rigid structure [56, 57]. Rutile TiO_2_ nanorods can also be precisely fabricated by a hydrothermal method (at 180 °C) and controlled by adjusting the amount of HCl and ethanol during the reaction [2, 58].

### Nanofibers

To fabricate ultrasensitive diagnostic devices (i.e., capturing cancer cells) or tissue regenerations, TiO_2_ nanofibers are one of the best candidates due to an improved local topographic interaction between the deposited nanofibers and extracellular matrix [59]. To produce long, fibrous nanomaterials, the electrospinning technique has been developed using a precursor, binder, and stabilizer [59]. Compared to the electrospinning strategy, other strategies, including self-assembly and template-assisted approaches, have proven unwieldy for the generation of TiO_2_ nanofibers [2]. Their diameter can mainly be altered by adjusting the diameter of the spin orifice, the conductivity, and the viscosity of the binding polymers as well as solvents [60, 61]. Employing a volatile solvent and less of the binder can further facilitate the removal of any residual organic substances from the final nanofiber structures [60]. Fabrication of porous and hollow TiO_2_ nanofibers is also important for different biomedical applications such as bone regeneration. In this case, a polymeric sol–gel solution composed of CaCO_3_ and TiO_2_ precursor that flows through a coaxial nozzle electrospinning into a cross-linker solution, produces CaCO_3_/TiO_2_ nanofiber, and the subsequent etching of CaCO_3_ on the calcined TiO_2_ fibers using dilute HCl fabricates porous and hollow TiO_2_ nanofibers [9]. Moreover, highly porous TiO_2_ nanofibers with a surface area of about 128 m^2^ g^−1^ can be generated using an electrospinning under high humid environment without applying a secondary chemical process or removal of the glycerin component [62].

### Nanowhiskers

Whiskers are one of crystalline materials with a distinct crystal anisotropy and possess high strength and fracture resistance close to the theoretical ultimate strength of a given material, whereas their size and length are smaller than short fibers. One-dimensional TiO_2_ nanowhiskers exhibit the highest photocatalytic efficiency due to a unique morphology and monocrystalline defect-free lattice structure in comparison to nanoparticles [5]. To produce TiO_2_ nanowhiskers, a reactant containing TiO_2_/K_2_O needs to be sintered at 810 °C, and then, the interim product should undergo a wet grounding. The potassium-rich nanophase gradually forms during a long incubation of the interim product in water (~ 7 days), and further HCl treatment and calcination generate a tetragonal crystal structure (anatase) [4, 5]. Rutile TiO_2_ nanowhiskers with diameters of ~ 10–50 nm and lengths of several micrometers can also be synthesized by annealing a precursor powder, in which NaCl and Ti(OH)_4_ particles (through an adjusted molar ratio) are homogeneously mixed [63].

### Nanotubes

One-dimensional TiO_2_NSs offer specific properties, including quantum confinement effects, electron tunneling, as well as a high surface area, draw exclusive attention to biomedical applications (i.e., drug delivery systems and biosensors) [64]. A vast number of strategies, including electrochemical anodization and hydrothermal, sol–gel, and electrospinning methods, have been exploited to fabricate TiO_2_ nanotubes.

While the fabrication of TiO_2_ nanotubes through bottom-up processes may be complex, variable, and low-yield, a cylindrical structure accompanied by a pure crystalline phase can be achieved [65, 66]. The self-organization of nanotubes through an alkaline treatment of TiO_2_ or titanium alkoxide powder can generate an anisotropic and open-end structure [67, 68]. Conversely, the mechanism involved in the hydrothermal method initially forms nanosheets, and a subsequent neutralization step triggers a rolling procedure to generate TiO_2_ nanotubes. The hydrothermal method is cost-effective due to the unprocessed metallic titanium source; however, the high concentration of the alkaline solution can often lead to excessive intercalation, thus assembling disordered nanotubes [67].

To fabricate uniform and ordered TiO_2_ nanotubes, template-assisted methods as an interesting alternative can be employed by depositing titanium oxide components on the outer or inner wall of nanoporous templates; the former is called a positive template, and the latter a negative template [69]. In both cases, a uniform, cylindrical structure made of either soft or hard anisotropic templates, including anodic aluminum oxide membranes (consisting of an array of monodisperse pores), multi-walled carbon nanotubes, soft polymeric templates and well-ordered zinc oxide nanorod arrays, can be employed to fabricate well-ordered TiO_2_ nanotubes [69–71]. The outer diameter and length of templates primarily determine the inner diameter and length of the final tubular structures, which can be open- or closed-ended [64]. Mesoporous TiO_2_ nanotubes can also be fabricated using a template-assisted method mediated with a sol–gel, followed by the calcination and template removal procedures [2, 45]. Although the magnitude of the specific surface area generally depends on the tubes’ length and diameter, features offered by mesoporous TiO_2_ nanotubes can be adjusted to the requirements of the specific biomedical application [45, 64]. Although template-assisted strategies certainly offer a wide variety of tubular structures and properties, their intractability restricts fine-tuning the final diameter, length, and order of TiO_2_ nanotubes.

**Fig. 2:**
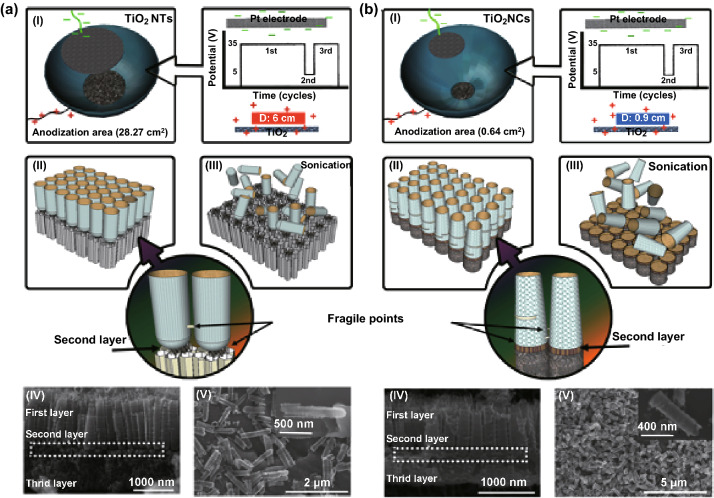
Schemes and representative SEM images of separated anodic TiO_2_ **a** nanotubes and **b** nanocylinders fabricated by using electrochemical anodization. The fabrication processes of the nanotubes and nanocylinders depend on the size of anodization cell (the area exposed to the electrolyte) and quick voltage changes (first cycle: 35 V and 240 min, second cycle: 5 V and 10 min, and third cycle: 35 V and 60 min). Adapted from Ref. [10] with permission from the American Chemical Society

To achieve well-ordered and well-aligned TiO_2_ nanotubes with a high aspect ratio, the electrochemical anodization of titanium foils in the presence of fluoride-rich electrolytes has proven practical [72, 73]. As depicted in Fig. 1c, electrochemical anodization offers systematic control over the morphology of TiO_2_ nanotubes by adjusting certain parameters, including viscosity, pH, fluoride concentration, stirring effect, temperature, water content of the electrolyte, anode–cathode working distance, potential applied, and processing time [74, 75]. To generate highly smooth nanotube arrays, for instance, an electrolyte composed of glycerol and fluoride ions can be employed in a longer period of processing time [10, 21, 76]. The fabrication of freestanding tubular membranes, contrarily, is possible through sonication and post-treatments (i.e., diluted hydrofluoric acid, hydrogen peroxide, and oxalic acid) [75]. Recently, we have expanded the electrochemical anodization to fabricate individual anodic TiO_2_ nanotubes and nanocylinders through a controlled time-varying protocol. By assembling a two-electrode anodization Teflon cell (a mesh-type platinum counter electrode placed above the titanium surface) and adding the electrolyte composed of ammonium fluoride (0.27 M) in glycerol/water (60/40, v/v) solution, a controlled three-step anodization (consisted of (i) 35 V for 240 min, (ii) 5 V for 10 min, and (iii) 35 V for 60 min) can precisely generate weak points at the multilayer tubular array, which can break into individual tubes by using mild sonication (Fig. 2). The adjustment of the physiochemical features, such as the electrolyte composition and voltage applied, is also required to trigger the generation of individually separated TiO_2_ nanotubes or nanocylinders on arrays by means of mild sonication. Moreover, the size of the area exposed to the electrolyte is critical for the formation of either TiO_2_ nanotubes (open on one end) or nanocylinders (open on both ends). For example, the use of the bigger exposed area (28.27 cm^2^) to the electrolyte fabricates TiO_2_ nanotubes, whereas the small area (0.67 cm^2^) produces nanocylinders at the same anodization conditions [10]. In another study, an indirect fabrication based on a bamboo-splitting mechanism (electrochemical anodization) has also been introduced for forming nanowires on the array, using a long-term anodization of titanium foils in the presence of a viscous electrolyte containing fluoride ions [77]. However, the fabrication of well-ordered and -aligned nanowires, nanorods, and nanoribbons has yet to be developed by means of electrochemical anodization.

### Nanosheets

Quasi-two-dimensional nanomaterials can play a critical role in biomedical applications as a result of their interfacial and mechanical properties [78–81]. TiO_2_ nanosheets can be fabricated through bottom-up strategies, including the hydrothermal approach, liquid-phase exfoliation, and self-assembly [79, 82]. In most cases, TiO_2_ nanosheets have been fabricated using the hydrothermal strategy based on the starting materials (i.e., tetrabutyl titanate) and high concentration of hydrofluoric acid as a capping agent in an autoclave at 200 °C for 24 h. Zhang and co-workers fabricated TiO_2_ nanosheets with the hydrothermal approach by adding 48% hydrofluoric acid dropwise into the titanate isopropoxide kept in a heated Teflon-lined autoclave chamber (180 °C) for 24 h [6]. Similarly, hexagonal titanate nanosheets with a tunable thickness and length can be generated by adding lactic acid [79, 83]. During the last few years, TiO_2_ nanosheets with different methods (i.e., the bacteria-assisted synthesis of nanosheet-assembled TiO_2_ hierarchical architectures [84]) have been fabricated, but these developed nanosheets have not been verified for biomedical applications.

## Post-fabrication of TiO_2_ NSs

### Crystalline Structure

In most cases, fabricated TiO_2_ NSs are amorphous and require additional thermal treatments to achieve a single or polymorphic crystalline structure. The crystalline structure of TiO_2_ NSs directly influences the photocatalytic activity upon UV irradiation. For example, TiO_2_ films with crystalline phases (anatase and a mixture of anatase and rutile) can generate higher amounts of reactive oxygen species (ROS) compared to the rutile phase [39]. A wide range of annealing temperatures can form different crystalline structures including anatase, brookite, rutile, and polymorph. Brookite crystals are always within crystalline TiO_2_ NSs, and pure brookite crystalline structures can be generated using the hydrothermal strategy [31, 32, 57, 85]. Annealing temperatures between 280 and 800 °C first create a polymorphic structure, and raising the annealing temperature toward 900 °C then increases rutile crystals within the polymorphic structure [74, 86]. Note that the annealing process in a vacuum or gaseous atmosphere, including nitrogen, argon, and nitrogen/hydrogen, also generates different polymorphic structures [74]. Interestingly, bottom-up strategies are able to directly synthesize crystalline structures consisting of different proportions of polymorphism compared to the top-down strategies [67]. On the other hand, the annealing process can be a major drawback with mesoporous TiO_2_NSs due to the pores potentially collapsing, their specific surface area being reduced or other properties changed (i.e., hydrophobicity). These changes may directly impact a number of biological responses, including cellular adsorption, interaction, and adhesion [87].

### Doping

The insertion of electronically active atoms into the lattice of TiO_2_NSs is an astounding strategy to engineer the original band gap (between 1.8 and 4.1 eV) for generating ultrasensitive biosensors and elaborated optical devices [31, 32]. Both transition-metallic (i.e., gold, platinum, iron, silver, lithium, and copper) and nonmetallic ions (i.e., nitrogen, carbon, fluoride, and sulfur) can be incorporated into TiO_2_ NSs to improve the valence and energy bands [88]. The doping can be performed through: (i) fabrication process into a solution composed of doping elements, (ii) thermal treatment in the presence of gaseous doping elements, (iii) ion implantation, (iv) anodic formation of alloys containing the transition-metallic elements, and (v) electrochemical doping approaches [32]. These doping strategies incorporate elements into the substitutional and interstitial sites of the lattice [89]. The most well-known doping elements (i.e., nitrogen, carbon, and sulfur) narrow the optical band gap by improving the valence band. The calcination of TiO_2_ NSs fabricated through wet approaches (precursors composed of glucose and tetrabutylammonium hydroxide) also generates a well-incorporated carbon doping within the structure [31, 90]. The other doping elements such as silicon, chrome, vanadium, and nickel incorporated into TiO_2_ NSs using an ion implantation can also improve the optical band gap. Although doping of TiO_2_ NSs can be performed through both wet and dry strategies, resulted properties are different. For example, nitrogen wet doping of TiO_2_ NSs quenches visible photocurrent and photocatalytic activities; however, these activities remain untouched by doping at the nitrogen/argon or ammonia atmosphere [31].

### Deposition

Another alternative strategy to lower the original band gap (e.g., n-type TiO_2_ semiconductor, ~ 3.2 eV) is the incorporation of metallic nanomaterials with a desired band gap into TiO_2_ NSs, to significantly improve optical, electronic, and catalytic properties. A wide range of strategies have been developed to deposit and coat (core–shell nanomaterials) different metals (i.e., platinum, gold, or silver) as well as quantum dots onto the TiO_2_ NSs. In addition, deposition of the nanostructured materials can remarkably affect cellular behaviors and responses, recognition of biomolecules, and ions at the interface [91]. For example, a simple deposition of gold nanoparticles on the surface of TiO_2_ nanotubes can significantly improve the glucose detection [92]. Deposition strategies, ranging from electrodeposition, chemical bath deposition, and the hydrolysis of precursors, have been developed to randomly decorate or fill TiO_2_ nanomaterials [93, 94]. Porous gold nanoparticles, for example, can be incorporated into TiO_2_ nanotubes by using combined approaches, including sputtering, dewetting, and etching [95]. To generate one-dimensional magnetic TiO_2_ nanomaterials, anodic TiO_2_ nanotubes can be soaked into a magnetic solution (i.e., ferrofluids) and the magnetic nanoparticles from the solution can be deposited on the tubes through an external magnetic field placed at the bottom of the tubular array. The fabricated magnetic anodic TiO_2_ nanotubes have a potential for being loaded with different therapeutics and guided with a magnetic field (i.e., a permanent magnet and magnetic tweezer device) to a target tissue [21]. In the case of nanofibers, TiO_2_-based precursors can be mixed with metallic nanoparticles in order to be easily incorporated into TiO_2_ nanofibers through the electrospinning technique [2].

### Self-Assembled Monolayers and Carbonization

A self-assembled functional monolayer on the surface of TiO_2_ NSs can lead to a selective conjugation of biomolecules, including proteins, ligands and antibodies, as well as the adhesion of mammalian cells. The formation of self-assembled monolayers on the surface of TiO_2_ NSs is more or less limited to carbonyldiimidazole, phosphonic acid, and organosilane-based reactive components [96]. Salonen and co-workers have also introduced a functional combination of carbon into the lattice and onto their surface to improve bioactivities [97, 98]. Hydrocarbonization process, an indirect short incubation of TiO_2_ nanomaterials in the presence of acetylene gas at high temperature (i.e., 850 °C), creates a graphitic monolayer on the surface to improve the mechanical stability [98]. The structure of the carbon monolayer in titanium oxycarbide depends on the incubation time and temperature. A hydrothermal reduction in graphene oxide can also warp the carbon monolayer on the surface of TiO_2_ nanoparticles [99]. A thermal annealing of the surfactant-coated nanomaterials (i.e., nanosheets) is an interesting alternative for the carbonization [100].

### Polymer and Biomolecule Conjugation

Bioconjugation strategies are an essential step for clinical translation of TiO_2_ nanomaterials in order to detect, track, visualize, target, and treat a wide range of diseases. To generate smart and flexible nanocarriers, polymeric coverage can impart a broad spectrum of new properties to TiO_2_ NSs. A thermo-, pH- and enzyme-responsive coverage can create smart, multistage theranostic nanoplatforms. A wide range of synthesized or natural polymers, including chitosan, polyethylene glycol, and polydopamine, have been employed for the conjugation, coating, and capping of TiO_2_ NSs [101–103]. Certain biochemical linkers, developed to temporary conjugate therapeutic agents, can be cleaved in a specific physiochemical condition by enzymes, irradiation, and the physiological environment (i.e., acidic pH of the endocytic compartments) [17]. In addition, the conjugation of biomolecules such as proteins, enzymes, and antibodies on the surface of nanomaterials plays a critical role in facilitating the detection of specific cells and therapeutic delivery to intracellular compartments, while reducing the risk of macrophages [104]. The biomolecules can be conjugated on the surface of TiO_2_NSs through various functional chemical linkers that can provide a rapid conjugation strategy to limit any bioactivity losses [96, 102]. Polymers, antibodies, and therapeutics can be conjugated through one of the following strategies (see *Bioconjugate Techniques* [105]):

- *Carbodiimide chemistry* A specific and practical conjugation strategy binds the primary amines of biomolecules and polymers to the surface of carboxyl-reactive TiO_2_NSs by means of the water-soluble 1-ethyl-3-(-3-dimethylaminopropyl) carbodiimide hydrochloride (EDC for an aqueous synthesis) and water-insoluble dicyclohexyl carbodiimide (DCC for an organic reaction). The carbodiimide coupling reaction at the physiological pH is less effective compared to the most efficient coupling condition at the acidic pH (~ 4.5), a simultaneous use of *N*-hydroxysuccinimide (NHS) or water-soluble Sulfo-NHS, and EDC is therefore recommended to provide the highest coupling efficiency at the physiological pH [106].
- *Click chemistry* A highly selective, high yield, and fast coupling reaction occurs between copper-catalyzed Huisgen cycloadditions of azides and alkynes to conjugate biomolecules, fluorophores, therapeutics, as well as polymers on the surface of TiO_2_ nanomaterials [107]. The surface of biomolecules and TiO_2_ nanomaterials can be modified by either the azide- or the alkyne-reactive moieties for the click coupling chemistry. However, a copper-free click chemistry is a point in order to eliminate the cytotoxic effects of the copper catalysts in biomedical applications. The activated biomolecules by means of a cyclooctyne (i.e., dibenzocyclooctyne = DBCO) can also bind to azide-labeled TiO_2_ nanomaterials.
- *Maleimide chemistry* Sulfhydryl-reactive chemical groups (–SH, thiols) are the most common cross-linker moieties for the conjugation of biomolecules on the surface of nanomaterials. In most cases, maleimide groups can specifically react with sulfhydryl groups at pH between ~ 6.5 and 7.5 to form a stable and irreversible thioether linkage. The coupling by the sulfhydryl groups is more selective and precise due to their limited available number on the biomolecules [108]. However, sulfhydryl-reactive chemical groups can be easily added through a reaction with available primary amines using the Traut’s reagent. Interestingly, plasmonic-deposited or -coated TiO_2_NSs can also be directly conjugated through a reaction with sulfhydryl-reactive chemical groups of the biomolecules to generate a bio-monolayer on the surface [109]. Moreover, the reduction in antibodies (i.e., using Tris(2-carboxyethyl)phosphine hydrochloride to cleave disulfide bonds) can expose their free sulfhydryl-reactive chemical groups to plasmonic- or maleimide-modified surfaces and create a direct conjugation [110].
- *Hydrazide*-*reactive chemistry* Targeted drug delivery based on antibody (an affinity-based binding) for the detection of exposed antigens, e.g., of cancer cell population, requires a precise conjugation strategy to less disrupt the Fab region of the antibodies. The oxidation of the carbohydrate at the Fc region of the antibody by using sodium periodate generates aldehyde groups, which can bind to the hydrazide moieties at the surface of nanomaterials [111]. The use of the hydrazone linkage can also create pH-cleavable linkers for certain biomedical applications (i.e., intracellular therapeutic delivery systems) in order to target specific subcellular compartments. Through a reaction between a carbonyl-reactive group (i.e., anticancer doxorubicin drug) and hydrazide moiety (i.e., on the surface of TiO_2_NTs) a pH-cleavable linkage can therefore be generated [10].

## Biological Responses to TiO_2_ NSs

### In vitro Cytotoxicity Assessments

The fundamental evaluation of potential health hazards caused by exposure to nanomaterials is now a crucial step. At the nanoscale, the size reduction in nanomaterials can trigger an excessive cellular uptake and subcellular accumulation and may disrupt activities of organelles [44, 97]. Transporting nanomaterials across the plasma membrane and accessing subcellular compartments rely on a multitude of factors, spanning surface properties and bioconjugation to size and morphology [112]. Internalization is initially affected by the cellular interactions between receptors located on the membrane and the surface of nanomaterials. Therefore, the communication between the cell receptors and nanostructure activates multiple endocytosis pathways, including clathrin-mediated endocytosis, caveolae, micropinocytosis, and phagocytosis [113]. Both aggregated and agglomerated TiO_2_ nanoparticles can be internalized into cells by phagocytosis, and monodispersed TiO_2_ nanoparticles can mainly be internalized through an energy-dependent endocytosis [114, 115]. Although the energy-dependent mechanisms are highly active for the endocytosis (e.g., of nanowires), the internalization efficiency depends on the aspect ratio of one-dimensional nanomaterials [116]. The rate of cellular uptake can be boosted through post-fabrications and surface modifications. A common size-dependent intracellular mechanism and localization roughly show an intracellular trafficking pathway, mostly ending up in endosomes and lysosomes [117, 118].

The incubation of TiO_2_ nanomaterials can induce both cytotoxic and genotoxic effects on mammalian cells by disrupting mitochondrial membranes [119, 120]. The toxicity can become exacerbated by increasing the dosage of TiO_2_ nanomaterials [121]. TiO_2_ NSs can primarily cause the production of ROS, DNA fragmentation, and oxidative stress and lesions (i.e., rendering nucleotides and inactivating base excision repair pathways) [122, 123]. The production of ROS, DNA damages, and chromosomal aberrations can arrest the cell cycle and subsequently trigger apoptosis [124, 125]. TiO_2_ nanoparticles can also cause a structural damage (i.e., mitochondrial damage and downregulation of ERK-pathway-related factor proteins), reduce the cell activity, and disturb the testosterone generation or secretion in the treated Leydig cells [126].

Bioactivity of nanomaterials is shape- and length-dependent. Cylindrical TiO_2_ nanomaterials, for instance, can induce significant apoptosis compared to spherical NSs [69, 127]. It was also observed that one-dimensional TiO_2_ nanomaterials can accelerate the formation of autophagosome-like vacuoles and the reduction in the mitochondrial calcium concentration [128]. Correspondingly, long TiO_2_ nanofibers can also disturb the transepithelial electrical resistance and perturbation, and generate a significant hemolysis and macrophage activation [127]. Anodic freestanding TiO_2_ nanotubes, for example, can also induce genotoxic cellular responses, including ROS production, without a significant cell death, while the non-anodic tubes are less toxic [125, 129].

The cellular responses to TiO_2_ nanomaterials can also be manipulated according to crystalline structures and surface chemistries in order to maximize cell viability and cellular uptake [130–132]. The super reactive crystalline structures induce a wide variety of toxicities related to the defect sites and distinctive crystal orientations [85, 133]. Both brookite and anatase TiO_2_ nanorods, for example, can reduce cell viability through the ROS production and expression of autophagosome proteins. However, an extensive distribution of lysosome and expression of endoplasmic reticulum proteins can be induced by anatase TiO_2_ nanorods [128]. On the other hand, polymeric surface modifications can reduce or diminish hazard risks caused by the administration of TiO_2_ nanomaterials [134–137]. The surface modifications may suppress the reactivity of crystals and minimize cellular and subcellular obstructive interactions [44, 125, 138]. However, polymeric surface modifications must be conscientiously optimized, because a hydrophilic and positively charged polymeric layer on the surface of nanomaterials may cause severe obstructive interactions within the subcellular compartments and consequently produce greater ROS and cytotoxicity [139].

The cellular responses are different toward one-dimensional arrays, such as implants and scaffolds with varying morphologies and structures [140–142]. The morphology, including pore size (or top-side diameter) and length of the tubular TiO_2_ arrays, mainly determines cell viability and proliferation through the modulation of the focal adhesion kinase and Ras homolog family member A (RhoA) pathways [35, 143, 144]. The macrophage inflammatory effect of tubular arrays is also controllable through the inhibition of mitogen-activated protein kinases and nuclear factor-κB pathways [145]. In addition, the change of the crystalline phase of tubular arrays can be another influencing factor in the adhesion and activation (e.g., platelets). The anatase nanotubes (annealed at 450 °C) can trigger the adhesion and activation behavior (i.e., spreading tendency and filopodia connections) of the platelets compared to as-formed amorphous nanotubes [66]. However, an elaborated surface modification including biomolecule conjugations alternatively improves cell viability, adhesion, and proliferation [144].

### In Vivo Inflammatory Responses

Partitioning of nanomaterials into organs and tissues occurs after entering into the cardiovascular system and may induce inflammation. The translocation of TiO_2_ nanoparticles depends on the injection site. Intravenous injection, for example, exhibits a high number of nanoparticles in the liver and a relatively smaller number in the spleen, lung, and kidneys [146–148]. Inhaled TiO_2_ nanoparticles that have quickly transferred into the circulatory system may randomly affect gene expression in the heart and lung [149]. TiO_2_ nanoparticles can then cause pulmonary inflammation through ROS production and the expression of inflammatory cytokines [150]. A recent study on the zebrafish exposed to TiO_2_ nanoparticles reveals no side effects on the hatching rate of zebrafish embryos and deformity; however, a long-term incubation of the nanoparticles with the adult zebrafish can cause an oxidative damage to the liver and gill (high expression of three antioxidant enzymes: superoxide dismutase, catalase, and glutathione S transferase) [151]. TiO_2_ nanoparticles can injure the liver through DNA breaks and chromosomal damages [147]. TiO_2_ nanoparticles localized in the spleen may cause apoptosis through the splenocyte dysfunction and proliferation of lymph nodules [152]. Moreover, TiO_2_ nanomaterials accumulating in the kidney can primarily cause severe dysfunction due to nephric inflammation and necrosis [147]. The translocation of TiO_2_ nanoparticles (5 nm in diameter, anatase) injected daily into the abdominal cavity for 2 weeks indicated the harmful results by triggering a consecutive series of intramolecular interactions, including a lipid peroxidation and a reduction in the capacities and functionalities of antioxidative enzymes in the brain [153]. Administering two-dimensional TiO_2_ NSs can also induce significant liver toxicity when changing the level of malondialdehyde, superoxide dismutase, and oxidative stress responses [6, 154, 155]. However, an elaborated surface modification may alter the partitioning or toxicity of TiO_2_NSs, thereby eliminating or reducing potential inflammation after their administration. Both freestanding anatase and brookite TiO_2_ nanorods, for instance, trigger immune responses and proinflammatory cytokines, but anatase nanorods cause fewer lesions compared to the brookite structure [128, 156]. In addition, normal spleen and thymus indexes without triggering immune responses after the administration of PEGylated TiO_2_ nanosheets, were reported [6]. The surface modification should also facilitate the clearance of the nanomaterials used for therapies. For example, after an intravenous injection, TiO_2_ nanoparticles (agglomerated with 73.3–95% of agglomerates with a peak size around 1400–1800 nm), which were quickly eliminated from blood and relocated in liver, spleen, and lungs, were interestingly cleared from the body with a half-life of 12.7 days [157].

## Biomedical Applications

### TiO_2_ NSs for Therapy

#### Therapeutic Delivery

Therapeutic vehicles based on TiO_2_ nanomaterials have been developed to deliver small molecules, proteins, and genes to target tissues and organs in the body. Increasing the surface area by generating pores within the nanomaterials maximizes therapeutic loading compared to their nonporous counterparts. As previously reported, the charge interactions between the therapeutic agents and nanomaterials mainly facilitate physical adsorption [158, 159]. In certain cases, the loading capacity can be alternatively increased by means of an external driving force such as voltage [160]. Apart from that, controlled release kinetics are also favorable for drug delivery systems. The release rate can be tuned using different strategies including polymeric capping or coatings on the surface of TiO_2_NSs [161, 162]. The controlled filling of nanotubes with drug-loaded polymeric micelles can generally slow down the release rate [163]. Drug reservoirs composed of TiO_2_ nanorods, nanofibers, and nanotubes also exhibit a sustained drug release for dermal drug delivery applications [164–166].

**Fig. 3:**
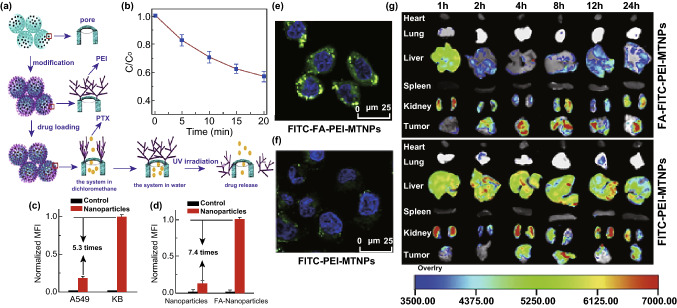
**a** Illustration of drug loading and the release process in multifunctional mesoporous TiO_2_ nanocarriers conjugated with PEI and folic acid. **b** The photocatalytic degradation of folic acid-PEI on the surface of mesoporous TiO_2_ nanocarriers through the use of UV light irradiation. **c** The improved cellular internalization of the nanocarriers into KB and A549 cells after 5 h (I) and the number of internalized nanocarriers with and without folic acid conjugation (II). FCM: Flow cytometry measurements. **d** Fluorescence images of treated KB cells after incubation (I) with and (II) without folic acid-conjugated nanocarriers. The punctuated green colors indicate the nanocarriers and blue (color) shows the cell nuclei. **e** In vivo fluorescence images of tissue collected at different times (post-injection). Adapted from Ref. [8] with permission from the Elsevier. (Color figure online)

A stimuli-responsive drug release for a precise chemotherapy in order to minimize side effects is achievable through the conjugation of pH-, thermo-, and enzyme-responsive polymers on the surface [44]. Multifunctional mesoporous TiO_2_ nanocarriers that had been conjugated with polyethyleneimine (PEI) and folic acid, for instance, were prepared for a drug delivery system based on the NIR laser-controlled drug release system [8]. X-ray illumination of TiO_2_ nanomaterials can create electron–hole pairs within the structure (degrading organic linkers) and generate a triggered release [18]. A combined strategy for a stimuli-responsive drug release, for example, has also been reported by allocating a hydrophobic cap for amphiphilic TiO_2_ tubular arrays sensitive to the UV light irradiation [17]. The multifunctional porous TiO_2_ nanoparticles, conjugated with PEI and folic acid, have also been developed for UV-responsive drug release as well as targeted drug delivery (Fig. 3a). The burst release of loaded anticancer drug paclitaxel from TiO_2_-based nanocarriers was controlled by the PEI capping, and the exposure to UV light irradiation, which accelerated the degradation of PEI on the surface by the generation of free-radicals, released the entrapped anticancer drugs (Fig. 3b). The improved cellular internalization of the folic-conjugated nanocarriers into KB cells (7.4 times higher than nonfunctionalized carriers) was obtained after 5 h of incubation (Fig. 3c). Compared to the treated KB cells (i.e., higher cellular uptake and cell death), the folic-conjugated nanocarriers induced less cytotoxicity in A549 cells due to the small cellular uptake (5.3 times less than that of KB cells) and demonstrated the selective cancer killing feature (Fig. 3d). In vivo fluorescence images of tissue, including the heart, liver, spleen, lung, kidney, and tumor, collected at different times (post-injection), also confirmed the improved cellular internalization and accumulation of the folic-conjugated nanocarriers into the target tumor after 4 h post-injection (Fig. 3e) [8]. In addition, PEI on the surface of the nanocarriers is also able to be swollen at the acidic pH of the intracellular compartments to cause a proton-sponge effect, which allows the cargo to be delivered to the cytoplasm [8, 19].

Targeted therapeutic delivery system is a key approach to accumulating therapeutics into the site of action in order to boost the therapeutic efficacy. The post-fabrication of nanocarriers by using biomolecules and ligands (i.e., folic acid, hyaluronic acid, and antibody) is a promising strategy, which can precisely accumulate nanomaterials at a specific tissue [8, 167, 168]. For example, the conjugation of CD133 monoclonal antibodies on black TiO_2_ nanoparticles to target the transmembrane glycoprotein highly expressed at pancreatic cancer stem cells has been developed to guide the nanoparticles for a site-specific cancer therapy [12]. Moreover, folic acid immobilization on the surface of anticancer-loaded nanocarriers can effectively promote the cellular uptake through a receptor-mediated endocytosis [8]. The penetration of therapeutic agents and nanomaterials is limited in solid malignant tumors [44]. However, the enhanced permeability and retention (EPR) effects, which occur in solid tumors, permit the nanomaterials to gain access to the restricted microenvironments [44, 97]. Correspondingly, a successful delivery of DOX to the orthotopic breast tumor has been achieved by the administration of polyethylene glycol-coated TiO_2_ nanoparticles based on the EPR effects [169]. After a long circulation time, an intracellular drug release is also an advantage for provision of a sufficient therapeutic effect. Intercellular drug delivery systems can be obtained by using different cleavable linkers (pH-, thermo-, and UV light irradiation), which temporary bind therapeutics on the surface of nanocarriers. The NIR light has also been employed to trigger an intracellular DOX release from zwitterionic polymer-gated Au@TiO_2_ core–shell nanoparticles. The NIR light irradiation (at 635 nm) to the internalized nanocarriers caused an efficient cell death via the accelerated DOX release into the cytoplasm and nucleus in comparison to the treatment without the laser irradiation [170].

**Fig. 4:**
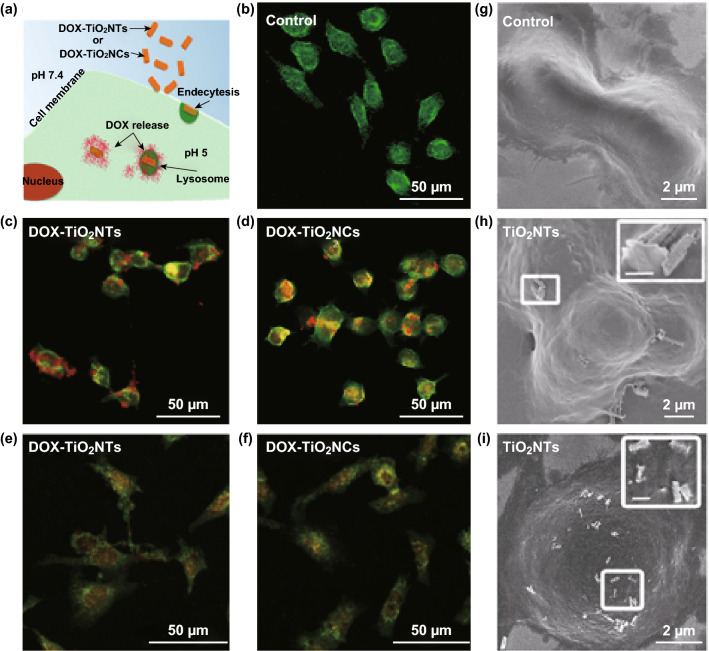
Internalization of DOX-loaded TiO_2_ nanotubes and nanocylinders into HeLa cells. (I) The cellular uptake and intracellular DOX release from TiO_2_NSs confirmed by scanning electron microscopy (SEM) and confocal microscopy images of control (-I-control) HeLa cells without the nanocarriers, DOX-conjugated TiO_2_NTs (-I-DOX-TiO_2_NTs, red demarcation) and DOX-conjugated TiO_2_NCs (-I-DOX-TiO_2_NCs, red demarcation) after 1 h and 24 h of incubation. Cells were stained with fluorescein diacetate (green). Insets indicate the magnified images of internalizing TiO_2_ nanotubes and nanocylinders. Scale bars of the inset frames are 500 nm. Adapted from Ref. [10] with permission from the American Chemical Society

Freestanding, one-dimensional TiO_2_NSs have been demonstrated to be remarkable platforms for drug delivery systems and cancer therapy. The shape of nanocarriers is a key parameter directly affecting circulation time, biodistribution, and cellular uptake in drug delivery systems. One-dimensional nanocarriers tend to adhere more to vascular endothelium compared to spherical nanocarriers, and improve endothelial targeting, e.g., of a solid tumor and intracellular localization [11, 44]. Recently, an intracellular DOX delivery has been developed by using individual anodic TiO_2_ nanotubes and nanocyliners to take advantage of a cleavable release based on a hydrazone linker in endolysosomes (Fig. 4). When the conjugated DOX molecules on the surface of freestanding TiO_2_ nanotubes and nanocylinders were exposed to the acidic environment (pH 5), the punctate red dots (related to DOX-loaded nanocarriers) were diminished and spread into the entire cell body. The endosomes with the acidic environment triggered the cellular death by cleaving covalently-bound DOX molecules from the nanocarriers. A short incubation (30 min) of nanocarriers (DOX-conjugated and DOX-loaded) with HeLa cells and the subsequent replacement of the culture medium (i.e., to eliminate all unbound nanocarriers and released cargo) indicated higher toxicity for the cells treated with pH-cleavable nanocarriers after 72 h, compared to the treatment with DOX-loaded nanocarriers [10].

**Fig. 5:**
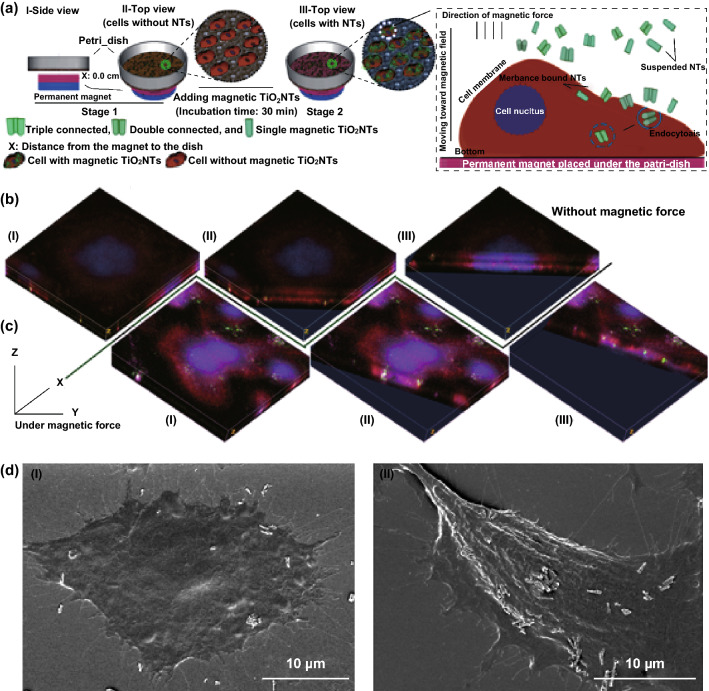
**a** Illustration of the improved delivery of anticancer drug camptothecin-loaded magnetic TiO_2_ nanotubes under a magnetic force. Representative confocal microscopy images (3D) and SEM images of enhanced cellular uptake **b** without and **c** with a magnetic field. The confocal images show magnetic TiO_2_NTs (green), actin filament (red) and nucleus (blue). **d** Representative SEM micrographs of internalizing magnetic TiO_2_NTs without (I) and with (II) magnetic force. Adapted from Ref. [21] with permission from the Springer Nature. (Color figure online)

Magnetically guided drug delivery systems possessing TiO_2_ nanocarriers functionalized with magnetic responsive materials can alternatively deliver therapeutics into the site of action. It might seem an impractical effort to employ an external magnetic force to target deep tissues under the skin (more than 5 cm) owing to a rapid reduction in the magnetic strength. However, it can be partially solved by implanting magnets in the body near the target site [171]. TiO_2_ nanomaterials are not susceptible to an external magnetic force, and in order to achieve this, the magnetic nanoparticles (i.e., iron oxide) can be embedded into the structure of the nanocarriers [159, 172]. For example, ferrofluid, a magnetic solution (composed of 3–15% iron oxide (magnetite) and 6–30% oil-soluble dispersant in 55–91% distillates (petroleum), viscosity of 6 mPa s, saturation magnetization 44 mT), can be incubated with TiO_2_ tubular arrays in order to deposit magnetic nanoparticles (~ 10 nm) and generate magnetic TiO_2_ tubular arrays. In comparison to relatively large magnetically guided TiO_2_ tubular arrays, their limited displacement at the target tissue, and the necessity for surgery to insert them into the body, freestanding magnetic TiO_2_ nanomaterials as an alternative can be potentially guided and accumulated into the site of action (Fig. 5). Moreover, magnetic nanotubes, which are sensitive to an external magnetic force (i.e., magnetic tweezer), can undergo displacements up to one micrometer (depends on the amount of deposited ferrofluid and position of nanotubes) after the attachment to the cells. A short exposure to the magnetic field can also improve the cellular binding, e.g., of the magnetic TiO_2_ nanotubes (~ 6 nanotubes per cell on average) and cause an enhanced delivery of anticancer camptothecin into the target cells (~ 90% killing efficiency) compared to the control groups without the magnetic force in vitro (~ 2 nanotubes per cell on average) [21, 173]. An increased deposition of magnetic nanoparticles into nanotubes might raise and improve the obtained small force (~ 2 pN) compared to commercially available magnetic beads (i.e., Dynabeads M-450 with ~ 54 pN force); however, a balance between the magnetic deposition and drug loading needs to be considered, without affecting the therapeutic efficiency.

Gene therapy as an efficient means of therapeutically delivering oligonucleotides can lead to curing and preventing a broad range of diseases. The short interfering RNA (siRNA) can be transferred to the intracellular compartments of targeted organs in order to silence specific messenger RNA [174, 175]. TiO_2_ nanoparticles have also been developed to deliver nucleic acid derivatives into the nuclei of target cells. Nanocomposite-based TiO_2_ nanoparticles and polylysine have been recently fabricated to deliver oligonucleotides as antiviral agents into nucleus of the Madin-Darby Canine Kidney cells. Oligonucleotide delivery to nuclei, found to be a cell division phase-dependent process, can happen during the interphase activity of cells, and the prophase condition mostly inhibits the internalization [176]. A sustained release of viral vector encoding proteins from the TiO_2_ tubular implants can diminish disadvantages of local delivery systems (i.e., short effective time, large dose requirement, repetitive administration, and poor distribution) [177]. To overcome these disadvantages, lentiviral vectors encoding BMP-2 loaded into the nanotubes by the lyophilization were released over 8 days and promoted osteogenic differentiation. This delivery system (lyophilization of the loaded vectors) has shown an advantage because of maintaining the stability of the vectors over the therapeutic period. Moreover, the sustained release of the lentiviral vector encoding BMP-2 improved the local cell accumulation by recruiting circulating bone marrow stromal cells around the TiO_2_ nanotubes and facilitated the differentiation into osteoblasts [177].

**Fig. 6:**
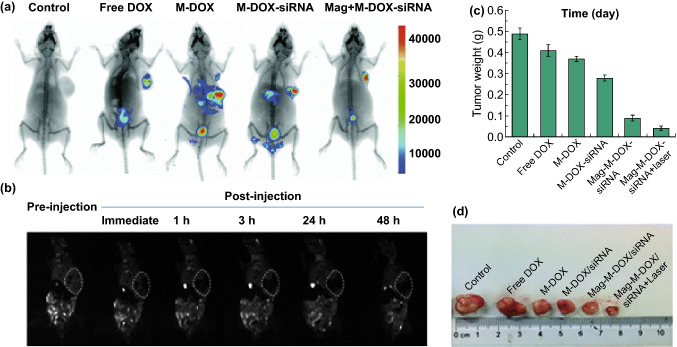
**a** Magnetic biodistribution of DOX/siRNA-loaded magnetic mesoporous TiO_2_ nanocarriers 12 h after administration in both control and tumor-bearing mice. **b** Captured T_2_-weighted MRI images during pre-injection and post-injection of the core–shell nanocarriers. Tumor regions are shown with the white, dashed circles. **c** The measured weight of the tumors of the treated and control mice. **d** Images of the tumors from the treated and control mice after the last injection (Abbreviation; M: Fe_3_O_4_@TiO_2_@mesoporous TiO_2_ and Mag: under a magnetic force). Adapted from Ref. [175] with permission from the Royal Society of Chemistry

A combined strategy for cancer therapy has been exhibited through the administration of TiO_2_-coated Fe_3_O_4_ core–shell nanocarriers loaded with doxorubicin (DOX) and β-catenin siRNA (Fig. 6). When mice treated with magnetic TiO_2_ core–shell nanoparticles were exposed to a magnetic force, a strong signal (T_2_-weighted MRI and fluorescence) was detected in the tumor site compared to the liver and other normal tissues within a fairly short period of time. The maximum accumulation of magnetic TiO_2_ core–shell nanoparticles in the tumors was reached at 3 h post-injection (based on T_2_-weighted MRI). The developed multifunctional carriers effectively silenced the β-catenin gene and caused remarkable apoptosis while suppressing proliferation. Exposure to NIR laser irradiation can also generate ROS and triggers tumor cell apoptosis in vitro and in vivo. The intracellular ROS and glutathione S-transferase level in treated cells by β-catenin siRNA- and DOX-co-loaded core–shell nanocarriers were intensively raised in a time- and laser-dependent manner (i.e., 3.7 W cm^2^ at 980 nm for 5 min) compared to the control groups in vitro. Moreover, the NIR irradiation showed higher ROS generation (3.74-fold) than that of the irradiated cells at 365 nm (UV region). The tumor volume of the mice treated with siRNA and DOX-loaded magnetic TiO_2_ core–shell nanoparticles (the effective co-delivery system) was dramatically reduced to 255 ± 32.2 mm^3^ compared to the control group (mean volume of 1914 ± 122.8 mm^3^), and the combined therapy exhibited 92.4% of tumor growth inhibition without inducing acute toxicity to the vital organs [175].

Alternatively, laser irradiation of gold-decorated TiO_2_ nanomaterials might be effective for the optoporation process. By using an ultrafast continuous or pulsed NIR laser irradiation of the nanomaterials bound on the cell membrane, optoporation can gently perforate the membrane lipid bilayer of target cells and internalize transgenes into the cytoplasm [178]. Compared to the nanocarriers, which are internalized based on the endocytosis mechanisms and need to trigger an endosomal escape pathway for releasing the transgenes into the cytoplasm, optoporation strategy can directly internalize the transgenes (i.e., mRNA and siRNA delivery) into the cytoplasm and accelerate either an expression or suppression of target proteins [19, 178]. For example, gold nanomaterials have been frequently used for the optoporation and subsequent delivery of siRNAs into the eye; however, this optoporation process is limited for the internalization of transgenes in deep tissues [179]. Therefore, a combined strategy based on laser and other treatments (i.e., ultrasound) may provide an adequate non-toxic energy and trigger plasmonic-modified TiO_2_ nanomaterials to perforate the cellular membrane in deep tissues for gene therapy.

#### Photo- and Sonodynamic Therapy

**Fig. 7:**
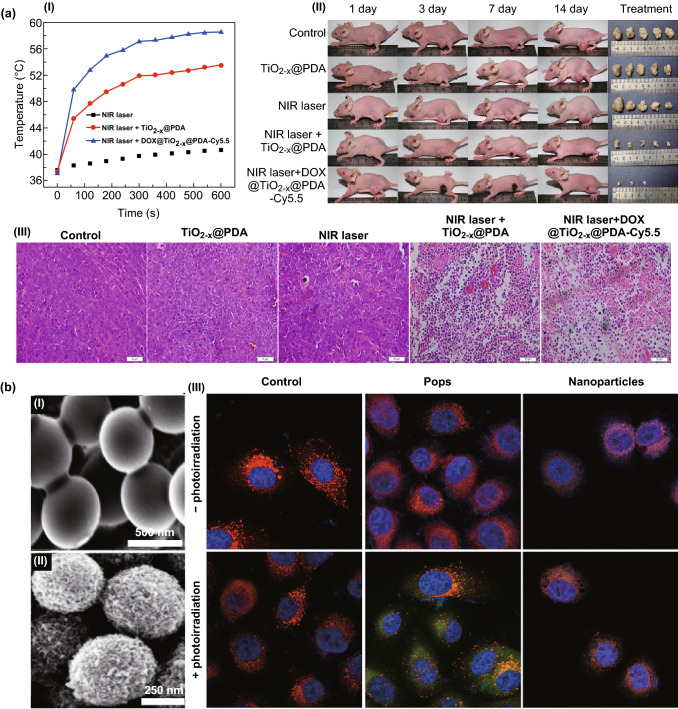
**a** Black TiO_2_ nanocarriers employed for a chemo/photodynamic/photothermal therapy in vivo. The efficient temperature raise (a-I) caused by black TiO_2_ nanocarriers (DOX@TiO_2−x_@PDA-Cy5.5) under NIR irradiation (808 nm, 1.0 W cm^−2^) triggers (a-II) a tumor growth inhibition. (a-III) Histological sections of the treated tumors indicate a noticeable cellular necrosis and apoptosis. Adapted from Ref. [47] with permission from the American Chemical Society. **b** Upon on/off-switchable photoactivation, TiO_2_ pops generate high-turnover, flash intracellular ROS. SEM images of mesoporous TiO_2_ Pops (b-I) before and (b-II) after solvothermal treatment. (b-III) The intracellular ROS generation using pops and smooth nanoparticles in the presence (+) and absence (−) of the irradiation. The green, red, and blue colors represent the intracellular ROS, mitochondria, and nucleus, respectively. Adapted from Ref. [15] with permission from the Springer Nature. (Color figure online)

TiO_2_ nanomaterials that are sensitive to two-photon irradiation generate remarkable amounts of the oxidative stress (ROS affects mitochondrial depolarization and caspase protein up-regulation) and induce hyperthermia that in turn initiates tumor cell apoptosis and necrosis [180]. For example, the hyperthermia effects appearing at temperatures higher than 46 °C can be generated by exposing the treated cells to NIR laser irradiation. Nevertheless, hyperthermia and ROS generation depend on the concentration, structure, geometry, and crystallinity of TiO_2_ nanomaterials employed [14–16]. Regardless of the photocatalytic efficiency and photostability of TiO_2_NSs, their low quantum yield is a significant drawback. Therefore, a lattice modification of TiO_2_ (i.e., conversion to the magnéli-phase Ti_8_O_15_) can significantly improve the originally low quantum yield and weak photodynamic properties [181]. Alternatively, a thermal hydrogenation of TiO_2_ NSs can generate black nanomaterials, with the presence of Ti_3_^+^ ions, oxygen vacancies, structural disorder/defects in the surface, Ti–OH groups, Ti-H groups, or modified valence band edge, which can improve photocatalytic activities [12, 13]. A short exposure of black TiO_2_ nanoparticles to a NIR laser irradiation can kill almost all treated cancer cells and significantly reduced tumor volume compared to the control groups in vivo [13]. The NIR irradiation-responsive drug release system based on black TiO_2_ nanoplatforms (DOX@TiO_2−x_@PDA-Cy5.5) has also been employed as a powerful strategy (a combined chemo/photodynamic/photothermal therapy) to inhibit the growth of breast cancer tumor in vivo (Fig. 7a). The DOX-loaded black TiO_2_ nanoparticles capped by polydopamine (1 mg mL^−1^) under NIR irradiation (808 nm, 1.0 W cm^−2^) were able to generate a temperature raise (ΔT up to ~ 24 °C) and caused a significant intracellular ROS generation and cell death (~ 95%) compared to each strategy carried out separately in vitro. Although the release of DOX from the nanocarriers is pH dependent, the use of NIR laser irradiation has additionally brought a switchable (on/off) release for the encapsulated DOX from the nanocarriers at both acidic and natural environments. The black TiO_2_ nanoparticles taking advantage of the combined chemo/photodynamic/photothermal therapies have also indicated a tumor growth inhibition feature in vivo, because those control groups receiving only photodynamic or photothermal therapy have met a poor therapeutic effect, and the irradiated tumors began growing 10 days after the treatment. The evaluation of histological sections of the irradiated tumor (treated animal models with DOX@TiO_2−x_@PDA-Cy5.5) also pointed out a massive cell necrosis and apoptosis compared to the control groups [47]. The biocompatible, mesoporous, TiO_2_ popcorn nanoarchitectures also offer a super-photocatalytic activity, which generates high-turnover, flash intracellular ROS (on/off-switchable photon-triggered ROS production) compared to smooth TiO_2_ particles (Fig. 7b). A solvothermal treatment (TiO_2_ beads mixed with ethanol/DI water (2:1 v/v) and 0.55 M ammonia solution and kept at 170 °C for 18 h) generates non-toxic TiO_2_ Pops (500 ± 50 nm in diameter and surface area up to 100 m^2^ g^−1^) with the anatase crystallinity and interacts much better with the complexity of the cellular membrane (i.e., lipid bilayer leakage) in comparison to the rutile structure. The intracellular ROS can be generated in prostate cancer cells (2.5-fold higher) by the photon excitation (3.5 mW cm^−2^ at 365 nm) of non-toxic TiO_2_ popcorn nanoarchitectures compared to the control groups without photoinduction [15].

A simple incorporation of noble metals into the surface of TiO_2_NSs alternatively improves quantum properties required for the generation of functional anticancer ROS [14]. For example, cobalt and nitrogen-doped TiO_2_ nanocrystals and TiO_2_-coated gold nanoparticles can enhance the photoactivation in the visible/NIR region [182]. An electrochemical deposition, e.g., of silver on the surface and a nitrogen doping of TiO_2_ nanoparticles can also change the treated cell morphology and increase ROS generation in human keratinocytes (HaCaT) and human lung epithelial cells (A549) and cause cell death via a late apoptosis/necrosis [183]. Due to a fast growth of new blood vessels for supplying oxygen and nutrients in tumors, antiangiogenesis is an advantage preventing the vascular growth and causes a significant tumor necrosis. Exposing tumors with internalized TiO_2_ nanomaterials to the second NIR irradiation (NIR-II) region (1000–1350 nm) can create efficient photoreactive effects against malignant tumors. TiO_2_-coated Au nanobipyramids, for example, have shown a high photothermal conversion efficiency up to ~ 93% (ΔT: ~ 27 °C) under NIR-II irradiation (at 1064 nm). The incorporation of anticancer combretastatin A-4 phosphate drug into the TiO_2_-coated Au nanobipyramids combined anticancer and antiangiogenesis activities has inhibited a lung tumor growth (0.4-fold smaller compared to the initial tumor size) in vivo [184].

Alternatively, ROS generation through the use of TiO_2_ nanomaterials sensitive to ultrasound has been recently improved as a means to kill cancer cells in deep tissues [185]. Au nanocrystals grown on the edge of the TiO_2_ nanosheets (band gap 2.90 eV) can induce an effective ROS generation through an ultrasound irradiation compared to pure TiO_2_ nanosheets (band gap 3.2 eV), and the engineered band gap prevents the fast recombination of excited electrons and holes that can improve the quantum yield of the ROS generation in vitro and in vivo [186]. The hydrophilized TiO_2_ nanoparticles activated by ultrasound can also generate ROS in the superficial tumors (i.e., intense vascular damage and proinflammatory cytokines) and suppress the growth of deep liver tumors (~ 15-fold) far more extensively than in the tumor-bearing mice without the ultrasound treatment [185]. In fact, sonodynamic therapy is limited to generating sufficient ROS against multidrug-resistant cancer due to the efflux of photosensitizer molecules from the P-glycoprotein [187]. Conjugating the trans-activator of transcription peptides on the surface of TiO_2_NSs can therefore overwhelm the effects of the P-glycoprotein and generate sufficient amounts of ROS, which directly breaks double-stranded DNA [187, 188]. Due to the nature of TiO_2_ nanomaterials sensitive to photo- and sono-dynamic therapy, a combined strategy may significantly improve the ROS generation and therapeutic efficacy.

### Medical Diagnosis

#### Bioimaging

Early-stage diagnosis and comprehensive understanding of diseases for employing an efficient therapy can be established through using ultrasensitive bioprobes [189]. A non-toxic surface modification by using a fluorescent molecule such as rhodamine B on the surface of anatase TiO_2_ nanoparticles can be the easiest option for the bioimaging of the target cells [190]. The sandwich-type electrochemiluminescence based on the tetragonal rutile TiO_2_ mesocrystals has also been developed to detect zearalenone, which is a mycotoxin secreted by Fusarium (human food contaminants). In most cases, a solution probe containing Ru (bpy)_3_^2+^ is incubated with an electrochemiluminescence setup to detect the target molecules; however, their immobilization on the surface of the TiO_2_ mesocrystals can amplify the emitted signal from the detected zearalenone [22]. However, long-term and real-time imaging, which is limited to conventional organic dyes and fluorescent proteins due to their short emission lifetimes, requires elaborated, stable bioprobes [44]. Recent studies also show that an extra doping procedure with gaseous nitrogen and other metallic elements generates photoluminescent TiO_2_ nanomaterials by changing the electron movement within the lattice and energy levels [191, 192].

**Fig. 8:**
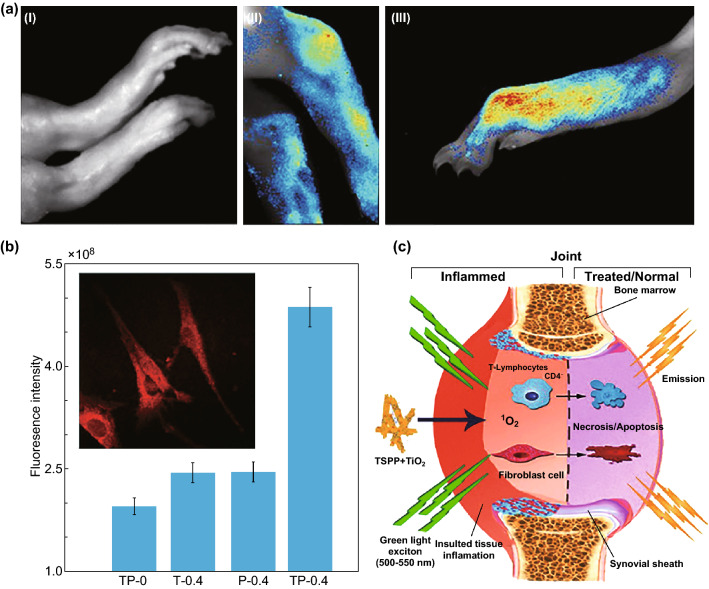
**a** Detection of rheumatoid arthritis using TSPP-modified TiO_2_ nanowhiskers; control sample (I), TP-0, shows no fluorescence; however, TP-0.4 (II) shows fluorescence at tibia-tarsal joint, and (III) in the infected foot. **b** The fluorescence intensities of different treatment groups illustrate the highest intensity for the group treated with TP-0.4. The inset shows fibroblasts from the rats’ RA joint with a bright red intracellular fluorescence. **c** Bioimaging and PDT properties of the TSPP and TiO_2_ nanowhiskers in the rheumatoid arthritis joint. The injection of 0.4 mL of TSPP-conjugated TiO_2_ nanowhiskers (TP-0.4), 0.4 mL of TSPP (P-0.4), 0.4 mL of TiO_2_ nanowhiskers (T-0.4), and control group without the injection (TP-0). Adapted from Ref. [23] with permission from the Springer Nature

Noninvasive detective and tracking strategies have been developed based on TiO_2_ nanomaterials in vivo. Tetera sulfonatophenyl porphyrin (TSPP)-conjugated TiO_2_ nanowhiskers, for example, have been developed as an effective fluorescence bioimaging and photodynamic agent for rheumatoid arthritis (RA) theranostics in vivo **(**Fig. 8). Fluorescence images can illustrate the tissue localization and cellular internalization of nanowhiskers in the RA synovium due to the excitation between 500 and 550 nm. An overview image of the infected feet and tibia-tarsal joint with the arthritis revealed a very strong fluorescence on day 16, and the exact location of the arthritis in the infected foot has been determined through imaging of the sagittal sections of the infected joints, which only emitted the fluorescence signal. These nanocomposites (TSPP-TiO_2_) can also produce singlet oxygens upon the green light excitation to necrotize the local cells (i.e., fibroblasts, and lymphocytes) due to an interference with the cellular pathways of adjacent cells. Although ^1^O_2_ lifespan of singlet oxygens generated within the tissue is short (~ 3 μs), the ROS resides for a longer period of time (~ 18 h) in the target tissue, efficiently causing apoptosis to the cells [23]. A simple surface modification (e.g., of gold grown on the edge of TiO_2_ nanosheets by using mitochondria-targeted triphenylphosphine and AS1411 aptamer) can enhance the computed tomography imaging of intracellular compartments due to the high atomic number of the grown gold nanomaterials [186].

Nuclear medicine imaging strategies, such as the positron emission tomography, are an alternative tool for a noninvasive detection and tracking in vivo due to their relatively long-time stability. For example, the incorporation of α and β emitters with TiO_2_ nanomaterials (i.e., ^48^V radionuclides) has been developed to generate supersensitive theranostic nanoprobes. The study on an animal model indicated a quantitative ^48^V TiO_2_ nanoparticles balancing of all organs (largely accumulated in the liver) without an interfering chemical background, and following the clearance process from 1 h to 4 weeks after the administration [193].

A magnetic resonance imaging (MRI) is a noninvasive clinical imaging technology, which has often been employed for disease diagnosis. In certain cases, MRI requires a contract agent, such as gadolinium (Gd), manganese, and iron oxide nanoparticles to enhance the visibility of tissues [194]. The development of MRI contrast agents based on Gd-enriched nanoprobes (i.e., enhance T_1_ MRI contrast) and superparamagnetic Fe_3_O_4_ and CoFe_2_O_4_ nanoparticles (i.e., improve T_2_ MRI contrast) is an advantage due to the adjustable conjugation of biomolecules on the surface, biodistribution, and magnetic property. A noninvasive tracing MRI agent, such as Gd-modified TiO_2_ nanoparticles, can visualize and verify the desired accumulation at the target tissue before triggering the release of cargo from stimuli-responsive nanocarriers and minimizing the side effects [24]. The biodistribution and accumulation of Gd- or Fe_3_O_4_-modified black TiO_2_ nanoparticles (high photothermal conversion efficiency) in the tumor can be precisely monitored for an effective photothermal therapy [12, 13]. Interestingly, the non-modified TiO_2_ nanoparticles (without adding magnetic or other contrast agents) can also improve MRI T_2_ proton relaxation time-weighted sequences as a contrast agent with the high concentration [25].

#### Biosensors

Diagnosing and monitoring diseases rely on the precise detection of biomolecules (proteins, genes, and cells, etc.) and can easily indicate a possible abnormality in the body. Both passive and active detection strategies must offer the ability to perform rapid in situ monitoring for health maintenance [195, 196]. Label-free TiO_2_ biosensors have been employed for the rapid detection of biological interactions converted to optical, electrical, and thermal signals. Amperometric biosensors, which consist of an oxidase and peroxidase, have proven critical in preventing issues related to enzyme instability and degradation [197, 198]. Enzymatic biosensors composed of an immobilized Prussian blue and an enzyme glucose oxidase on the surface of gold/TiO_2_ tubular nanocomposites have exhibited a rapid responsiveness, wide linear range, and stability [92]. Conversely, optical interferometric and surface-plasmon-based biosensors have been successfully used to design label-free TiO_2_ biosensors. For example, stable TiO_2_ nanotubes fabricated to sense rabbit immunoglobulin G (IgG) with optical interferometry (reflective interferometric Fourier-transform spectroscopy) exhibited super-sensitivity as well as real-time detection [30]. In fact, the porous structure of TiO_2_ nanotubes has a strong impact on the sensitivity of biosensors due to higher surface activity and greater electron transfer rates. The mesoporous nanostructures (glucose oxidase electrode), easily coordinate amine and carboxyl groups on the surface, behave as an electron mediator and improve the electron transfer between the redox centers of the enzymes and the electrode surface [199].

Photoelectrochemical biosensors are the alternative detection device based on the charge separation and transfer upon illumination and are highly dependent on substrates as a photoactive material. Modified TiO_2_ tubular arrays, absorb and respond to the visible light and can also play a critical role in the generation of cost-effective ultrasensitive biosensors [200, 201]. The surface modification of TiO_2_ nanotube arrays by means of polydopamine can facilitate the horseradish peroxidase decoration for a quantitative H_2_O_2_ detection (range from 1 nM to 5 μM) combined with an enzyme-induced biocatalytic precipitation amplification [200]. The copper-doped TiO_2_-grafted C_3_N_4_ as a photosensitizer, for example, has improved the detection of the emitted signal from the alkaline phosphatase and catalyzed the ascorbic acid 2-phosphate to ascorbic acid as a direct electron donor (reduced background signal interference) [202].

An ultrasensitive photoelectrochemical cytosensing platform has been developed through an electrochemical reduction in graphene (EG)/ZnIn_2_S_4_-co-sensitized TiO_2_ and immobilization of phosphatidylserine-binding peptides to capture apoptotic cells. Compared to other assays, a stable and non-toxic photoelectrochemical cytosensing platform based on the reduction in the photocurrent signal can exactly detect and capture apoptotic cells (a linear range from 1 × 10^3^ to 5 × 10^7^ cells mL^−1^). This platform can also retain the normal cell growth and proliferation for further precise assessments of therapeutic effects [201]. A label-free microfluidic immunosensor with high sensitivity (a range from 1 × 10^−15^ to 0.1 × 10^−6^ M) and selectivity has also been developed for an early detection of epidermal growth factor receptor 2 (quantify breast cancer biomarkers) based on an immunoelectrode made of porous hierarchical graphene foam modified with electrospun carbon-doped titanium dioxide nanofibers (as an electrochemical working electrode). This porous hierarchical graphene foam composition with functional carbon-doped TiO_2_ nanofibers has shown an increased charge transfer resistance, surface area, as well as improved porous access to the sensor surface by the analyte [27].

The post-fabrication of TiO_2_ electrodes with receptors associated with targeted molecules makes field-effect transistor (FET) biosensors a versatile probing device. A real-time, selective, and sensitive FET biosensor accompanied by an electrode composed of TiO_2_ nanowires has been furthered for targeting IgG proteins at the nanogram level [203]. As a matter of fact, a contamination of biosensors in the non-labeled area can reduce the sensitivity of functional substrates. Biosensors also face the obstacle of remaining analytes making FET biosensors non-reusable after detection. However, a reusable FET biosensor based on TiO_2_ composites encapsulated in graphene oxide has been recently introduced for a protein detection without sensitivity losses [29]. The immobilization of monoclonal antibodies on the surface of TiO_2_ nanowire bundles can also create a microelectrode-based FET sensor for a rapid and sensitive detection of *Listeria monocytogenes* without interfering with other foodborne pathogens [204].

**Fig. 9:**
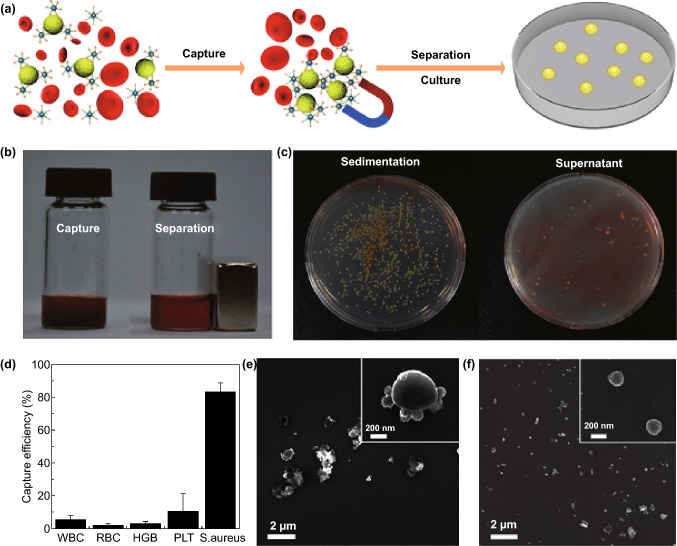
**a** The illustration represents the strategy for the identification and capture of pathogenic bacteria (*S. aureus*). **b** The photograph exhibits the capture (left) and separation (right) of bacteria with Fe_3_O_4_/TiO_2_ core–shell nanoparticles from an infected blood. **c** The number of colony-forming units in the re-cultured *S. aureus* from sediment and supernatant in agar plates after the separation. **d** Capture efficiency of different compounds in blood after being treated with Apt_S.aureus_-Fe_3_O_4_@mTiO_2_. SEM images of the captured *S. aureus* **(e)** and non-captured *E. coli* **(f)** with the aptamer decorated nanoparticles. Adapted from Ref. [28] with permission from the American Chemical Society

TiO_2_ nanofibers outfitted with cell-capture agents exhibit a remarkable ability to capture circulating colorectal and gastric tumor cells [108]. Moreover, gold-coated TiO_2_ butterfly-like three-dimensional membranes decorated by lectin molecules have demonstrated a selective recognition between highly invasive (T47D) and less invasive (MCF7) cancer cell lines [26]. Capturing the cancer cells in order to culture them for further investigations is an advantage that has been obtained by using gelatin film-coated TiO_2_ nanopillar arrays. Due to the high surface area and the interaction with the cell membrane’s antigens, the capture efficiency was achieved up to 94.98%, and a rapid digestion of the gelatin layer provided a nondestructive release of the captured cells for future proliferation [205]. Replacing the antibodies and other biomolecules with aptamers can be an alternative strategy for amplifying the detection sensitivity beyond that of conventional biosensors. Mesoporous TiO_2_-coated magnetic nanoparticles have been decorated with a sensitive aptamer to fabricate a pathogen capture platform in the blood stream (Fig. 9). The presence of aptamers and iron nanoparticles (core) facilitates the identification, capture, and separation of the bacteria. The conjugation of aptamer on the nanoparticles to detect *S. aureus* cells indicated that the capture efficiency of the platform was about 83%. Current strategy in clinics uses a continuous blood culture system, which is incubated with the blood sample collected from the patient, and the production of metabolite in the culture indicates possible bacterial infections in a time-consuming manner (up to several days). However, aptamer-functionalized nanoplatforms can precisely capture and then separate the target bacteria from the blood sample (using a magnetic force) with a negligible capture of other cells (i.e., white and red blood cells, hemoglobin, and platelets) (Fig. 9d). In addition, an efficient capture of *S. aureus* by the aptamer-modified nanoparticles was found, and highlighted this selectivity mechanism based on the creation of a sequence-defined unique structure (Fig. 9e, f). The inoculated bacteria on solid medium can be further used for different examinations such as colony counting, owing to quick enrichment within 2 h. The bacterial enrichment of clinical blood samples due to the high selectivity and strong affinity of the aptamers might be an elaborate strategy to regulate administration of an effective antibiotic therapy at an early stage [28].

### Tissue Regeneration and Chronic Wound Healing

**Fig. 10:**
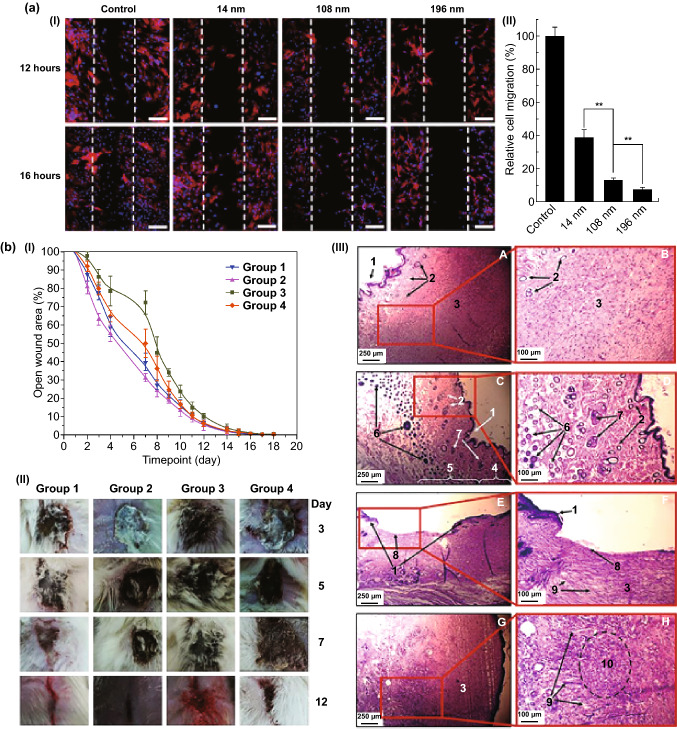
**a** Cellular migration of mesenchymal stem cells incubated with different sized TiO_2_ particles. Scale bars show 100 μm. Adapted from Ref. [208] with permission from the Dove Press Ltd. **b** The wound-healing process (I) macroscopically analyzed over the course of 19 days, (II) the representative wounds through the healing process for each group, and (III) the optical images of the healed skin from group 1 (A and B), group 2 (C and D), group 3 (E and F), and group 4 (G and H). Numbers indicate tissue structural elements: 1—epidermis; 2—sweat glands; 3—scar; 4—derma; 5—hypodermis; 6—hair follicles; 7—sebaceous glands; 8—de-epithelialized scar tissue; 9—scar vessels; 10—inflammatory infiltration in the scar. Adapted from Ref. [214] with permission from the Springer Nature

The human body’s self-healing process is slow when the injury is severe. However, the body indeed accepts external aids from implanted biological tissues and organs grown in the laboratory as a means to accelerate the healing process. It is crucial that scaffolds or implants in the body communicate with the surrounding microenvironment since the recipient’s immune system may very likely cause rejection. Biocompatible TiO_2_ nanomaterials are one of the greatest implantable materials for tissue regeneration owing to their properties of high tensile strength, flexibility, and corrosion resistance. The morphology of TiO_2_ nanomaterials (i.e., nanotubes) is, nevertheless, the most important factor in improving cell adhesion, proliferation, and differentiation [35, 36]. A scaffold composed of polylactic-*co*-glycolic acid (PLGA) and TiO_2_ nanoparticles as well as decorated glass with TiO_2_ nanoparticles can improve the amount of precipitated calcium for bone regeneration compared to the scaffold without TiO_2_ nanoparticles [206, 207]. The adhesion and spreading of osteoblast cells with a complete integration can also be attained with composites made of polylactic acid (PLA), poly-ε-caprolactone (PCL), and TiO_2_ particles or nanofiber meshes mimicking the bone regeneration properties [37, 38]. Compared to functionalized nanomaterials, it was observed that mesenchymal stem cells prefer to migrate without the interfering features of bare TiO_2_ nanoparticles. It was shown that bare TiO_2_ nanoparticles with different sizes can induce negative impacts on viability, adhesion, migration, proliferation, and differentiation of mesenchymal stem cells in a size- and dose-dependent manner in vitro; however, small bare TiO_2_ nanoparticles can activate the migration of mesenchymal stem cells compared to larger bare nanoparticles (Fig. 10a). The alkaline phosphatase activity, which determines an early mineralization-related protein marker for osteogenesis of osteoblasts, was also increased in the mesenchymal stem cells treated with TiO_2_ nanoparticles (14 nm in diameter) after 2 weeks compared to those treated with bigger nanoparticles (108 and 196 nm in diameter) [208]. Biomolecule-TiO_2_ nanohybrids can be an advantage due to improving antibacterial and -inflammatory features and biocompatibility without increasing the content of TiO_2_ in vital organs.

In the case of TiO_2_ tubular arrays, for example, the diameter, porosity, and curved surface regions of the tube directly affect cell viability and proliferation [35, 36]. Slight changes in the microenvironment can alternatively regulate osteoblast responses during integration of implants with host bones. Iron-doped TiO_2_ nanotubes (4.25 wt% Fe), for example, can alter the microenvironment and enhance the proliferation, gene expression of osteogenesis-related proteins, collagen secretion, and extracellular matrix mineralization of osteoblasts compared to as-formed tubular implants [209]. Icariin, a traditional Chinese medicine with a strong antiapoptotic ability in osteocytes and osteogenic function, can also be loaded into TiO_2_ nanotubular implant to obtain a slow release of the cargo (over 2 weeks) and promote osteoblast cell adhesion, proliferation, and differentiation in vivo [210]. Among other things, biomolecule coverage, which uses bone morphogenetic protein 2 and a peptide fragment of bone sialoprotein on the surface (e.g., TiO_2_ nanotubes and nanofibers), alternatively exhibits significant proliferation and osseointegration [211, 212]. Recently, a conjugation of sclerostin antibody on the surface of nanotubes, which stimulated Wnt signaling pathway by the reduction in the sclerostin secretion from MLO-Y4 cells (murine osteocyte-like cell line), also promoted the differentiation of osteoblasts in a co-culture [213].

The use of TiO_2_ nanoparticles can also effectively accelerate the wound-healing procedure in vivo for both second-degree and fourth-degree burns. Triggering factor XII (FXII) and contact system-triggered pathways can cause blood coagulation and clot formation for wound healing. Therefore, TiO_2_ sol sprayed on the burn wounds in rats adapted to a second-degree burn (groups 1 and 2, untreated and treated, respectively), and a fourth-degree burn (groups 3 and 4, untreated and treated, respectively) showed a boosted reduction in the exposed wound area (Fig. 10b). The treated wounds with nanoparticles (group 2) have revealed a healing outcome comparable to the normal skin conditions without showing a skin structure alteration, whereas the group 1 without the treatment was subjected to the thickened fibers lying tightly in the papillary layer with a reduced population of glands and flattened sweat epithelium. The activation of fibroblasts and overproduction of the basic substance were also observed at the reticular layer of these rats (group 1). Severe abnormalities (i.e., damaged epithelium and vascularization and increased fibrosis hypodermis) have been investigated for rats in the group 3 without the treatment. However, a daily base treatment using TiO_2_ nanoparticles for rats in group 4 brought obvious wound-healing effects by thickening epithelium layer, reducing dermal thickness, as well as increasing the formation of new blood vessels and base materials [214].

Nevertheless, either bone and wound infections or dental abscesses are highly likely because of possible contamination of implants and systemic diseases; infections can be minimized by the physical adsorption of anti-inflammatory drugs or silver nanoparticles onto TiO_2_ NSs [140, 215]. Moreover, it has been proved that antimicrobial activity of TiO_2_ nanomaterials eliminates infections and accelerates the proliferation of cells in the wound area compared to other materials [216–219]. For example, a mat composed of silk fibroin nanofibrous and TiO_2_ nanoparticles has been developed not only to improve the adhesion and proliferation of fibroblasts, but also trigger an antibacterial activity against *Escherichia coli* under UV light irradiation [40]. In another study, electrospun polyurethane membranes with TiO_2_ nanomaterials assembled in situ have exhibited an antibacterial effect in fighting *Pseudomonas aeruginosa* and *Staphylococcus aureus* and then caused a promoted adhesion of fibroblasts [220]. A quick UV light irradiation can activate bacteriostatic features in TiO_2_ nanomaterials and accelerate the wound-healing process [221]. TiO_2_ films with crystalline phases (anatase and a mixture of anatase and rutile) can produce higher amounts of ROS and biofilm reduction (composed of Streptococcus sanguinis, Actinomyces naeslundii, and Fusobacterium nucleatum) after the UV light activation compared to pure rutile TiO_2_ films [39]. Among metallic nanomaterials, silver nanomaterials are one of the well-known anti-infective agents used for wound dressing, but their resistance to silver is an emerging issue impairing the wound-healing process [222, 223]. Hence, a composition of TiO_2_ nanomaterials and antimicrobial polymers can improve the wound-healing process, because the antimicrobial activity of TiO_2_ nanomaterials under UV light irradiation can be harnessed against heavy-metal-resistant bacteria.

## Conclusions and Future Perspectives

TiO_2_ is a promising biomaterial for decoding a wide variety of limitations present in nanomedicine, and also thanks to its easy fabrication, post-fabrication, and biocompatibility. This review focused on the theranostic properties of TiO_2_ nanomaterials developed for a variety of unique and limited applications in nanomedicine. A broad range of TiO_2_ nanomaterials have been fabricated with high precision and post-fabricated with adjustable physiochemical properties. Biocompatible TiO_2_ nanomaterials are unique due to a wide range of features (i.e., a tunable geometry, dimension, porosity, as well as quantum effect, its photoactivity and well-established surface chemistry) that generate less toxic biological responses. There has also been substantial progress in fabrication and post-fabrication of TiO_2_ nanomaterials to obtain the best performance for different biomedical applications in vivo. However, to realize their theranostic potential and predict clinical outcomes, there are critical limitations and challenges that need to be addressed.

Many promising studies show the successful development of TiO_2_ NSs for therapies in vitro and in vivo; however, their translation into a clinical setting remains unexplored mainly due to long-term biocompatibility uncertainties. TiO_2_ nanomaterials are not biodegradable, and it is crucial to focus on procedures to accelerate their clearance after the therapies. Preclinical studies show that administered NSs can be diminished from organs for a period of 1 week to 1 month after the treatment without harm; however, the removal process in these cases proved to be size, shape, and dosage dependent [157, 224]. Therefore, an elaborated design of TiO_2_ nanomaterials based on biological microenvironments and responses is still needed to minimize long-term cytotoxicity and accelerate the clearance process.

Recently, significant advances have also been made with respect to sensitivity, specificity, and reproducibility, thus furthering real-time, wearable, and implantable TiO_2_ nanomaterial-based biosensors. Nevertheless, many challenges, including false detection from complex biological fluids, still remain and must be overcome for practical and clinical purposes. In doing so, the long-term stability of such bio-detectors has to be improved, biofouling diminished and supersensitive receptors integrated regardless of other interfering biomolecules.

Clinical-use implants based on orthopedic TiO_2_ nanomaterials, offering significant osseointegration and greatly imitating the strength of bone structure, are well known. However, cellular responses to nanoscale TiO_2_ biomaterials used to directly or indirectly support the cellular differentiation and proliferation are ambiguous due to an unknown long-term biocompatibility. A multicomplex nanohybrid system composed of smart biocompatible polymers and TiO_2_ NSs still needs to be developed to successfully regenerate and repair tissues, hereby extending human life. Our knowledge of the clinical potential of TiO_2_ nanomaterials in biomedical applications is extremely limited, and ongoing, comprehensive, and multidisciplinary studies are required to adjust the inherent properties of TiO_2_ nanomaterials.

All things considered, most of the studies as a proof of principle have demonstrated that TiO_2_ nanomaterials have the potential to overcome challenges in certain aspects associated with nanomedicine. An elaborated design of multifunctional TiO_2_ nanomaterials based on biological microenvironments and responses may improve the limited theranostic efficacy. Thus, further preclinical studies of functionalized TiO_2_ NSs still need to be taken into account in order to improve biological responses and to diminish side effects before these nanomaterials can be translated into clinical settings.
